# Establishment of a prediction model for histological chorioamnionitis and its association with outcomes of premature infants

**DOI:** 10.3389/fped.2023.1194563

**Published:** 2023-08-16

**Authors:** Li Zhang, Xin Fang, Zhankui Li, Xiang Han, Hongyan Du, Pengfei Qu, Feifei Xu, Lizhi Wu, Yajun Li

**Affiliations:** ^1^ Department of Neonatology, Northwest Women’s and Children’s Hospital, Xi’an, China; ^2^ Graduate School of Xi’an Medical University, Xi’an, China; ^3^ Department of Obstetric, Northwest Women’s and Children’s Hospital, Xi’an, China; ^4^ Department of Pathology, Northwest Women’s and Children’s Hospital, Xi’an, China; ^5^ Translational Medicine Center, Northwest Women’s and Children’s Hospital, Xi’an, China

**Keywords:** histological chorioamnionitis, premature infants, prediction, sepsis, infection

## Abstract

**Aim:**

This study aims to construct a prediction model for histological chorioamnionitis (HCA) and analyze the associations between the predicted risk of HCA and adverse outcomes in preterm infants.

**Methods:**

In total, 673 subjects were included in this cohort study and divided into HCA group (*n* = 195) and non-HCA group (*n* = 478). A stepwise method was used to screen the predictors for HCA, binary logistic regression was used to construct the prediction model, and the associations between the predicted risk of HCA and adverse outcomes were analyzed.

**Results:**

HCA occurred in 195 patients, accounting for 29.0%. The sensitivity of the prediction model was 0.821 [95% confidence interval (CI): 0.767–0.874)], the specificity was 0.684 (95% CI: 0.642–0.726), the positive predictive value was 0.514 (0.459–0.570), the negative predictive value was 0.903 (95% CI: 0.873–0.934), the area under the curve was 0.821 (95% CI: 0.786–0.855), and the accuracy was 0.724 (95% CI: 0.690–0.757). The predicted risk of HCA was associated with a higher risk of bronchopulmonary dysplasia (BPD) [odds ratio (OR) = 3.48, 95% CI: 1.10–10.95)], sepsis (OR = 6.66, 95% CI: 2.17–20.43), and neonatal infections (OR = 9.85, 95% CI: 3.59–26.98), but not necrotizing enterocolitis (OR = 0.67, 95% CI: 0.24–1.88), retinopathy of prematurity (OR = 1.59, 95% CI: 0.37–6.85), and brain damage (OR = 1.77, 95% CI: 0.82–3.83). After adjusting for confounders including gestational week at birth and birth weight, the risk of neonatal infections (OR = 5.03, 95% CI: 2.69–9.41) was increased in preterm infants’ exposure to HCA.

**Conclusion:**

The model showed good predictive performance for identifying pregnant women with a higher risk of HCA. In addition, HCA was associated with the risk of BPD, sepsis, and infections in neonates.

## Introduction

Chorioamnionitis (CA) is an acute inflammation of the amnion and chorion, serving as an indicator for intra-amniotic infection (1). CA is frequently asymptomatic and requires confirmation through histopathological examination of the placenta. HCA may be caused by bacteria, but some cases might be due to sterile inflammation (2). CA was reported to be associated with the increased risk of preterm birth, with 40%–80% of preterm deliveries being affected by this condition (3). It is divided into clinical CA (CCA) and histologic CA (HCA) (4). CCA has obvious clinical manifestations and is easy to be identified (5). Due to the insufficient recognition of HCA, it cannot often be timely diagnosed, which may result in a poor outcome of infants (6). It is imperative to promptly identify patients at high risk of HCA in order to prevent disease occurrence and enhance neonatal outcomes.

Strong evidence supported that HCA is associated with adverse neonatal morbidities in preterm birth including newborn respiratory distress syndrome (NRDS), sepsis, bronchopulmonary dysplasia (BPD), and death (7, 8). Watterberg et al. identified that HCA might reduce the risk of RDS (9). A meta-analysis indicated that preterm HCA may be a risk factor for cerebral palsy (10). Another study reported that HCA leads to a fetal inflammatory response syndrome that can reprogram the developing immune system and cause lifelong effects (11). Therefore, to prevent the occurrence of HCA is of great value for providing timely interventions to improve the outcomes of infants. Currently, several prediction models were established for the occurrence of HCA during pregnancy using some laboratory indexes including the neutrophil–lymphocyte ratio, maternal C-reactive protein (CRP), intercellular adhesion molecule-1 (ICAM-1), interleukin 6 (IL-6), interleukin 8 (IL-8), and matrix metalloproteinase 8 and 9 (MMP-8 and MMP-9) (12–15). The area under the curves (AUCs) of these models ranged from 0.70 to 0.89, but these studies were mainly focused on predicting HCA in preterm newborns with premature rupture of membranes during pregnancy, and the sample sizes were small. A more reliable model for predicting HCA in preterm newborns was required.

In the current study, the risk of HCA was predicted via constructing a prediction model, and the associations between the predicted risk of HCA and the adverse outcomes in preterm infants were analyzed.

## Methods

### Study population

**Figure 1:**
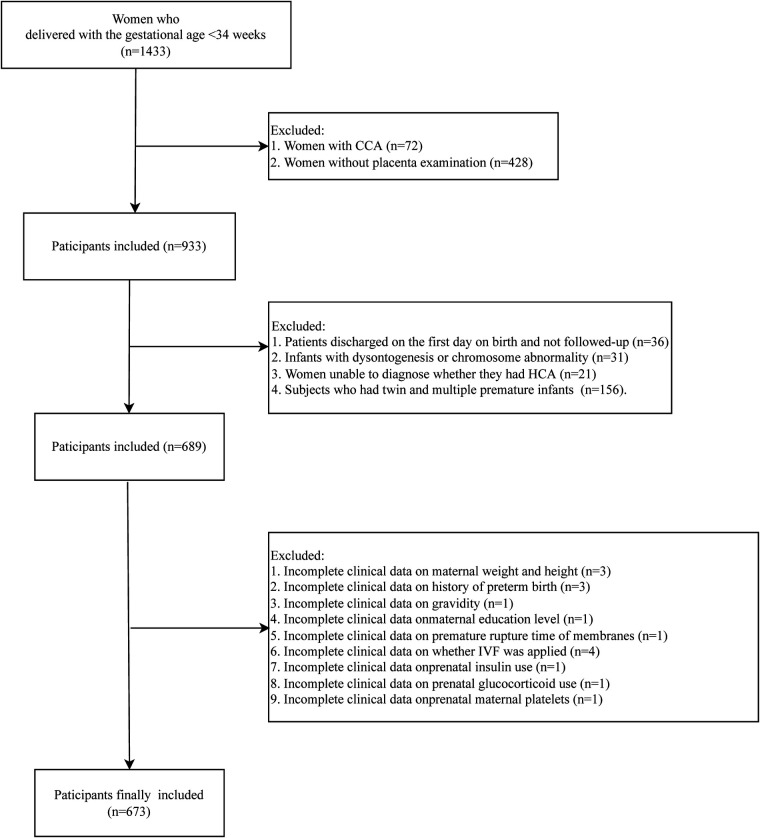
Screen process of the participants in this study.

In this retrospective cohort study, we retrospectively collected the data of 1,433 subjects who delivered in the Department of Neonatology, Northwest Women's and Children's Hospital, between 1 June 2018 and 31 May 2020. Participants who had singleton pregnancy with live birth whose gestational age was <34 weeks were included. All infants were admitted to the Neonatal Department during the neonatal period. Patients with CCA were not included in this study (*n* = 72). The pathology of placenta was examined in 933 subjects. Among them, 36 patients were discharged on the first day on birth and were not followed-up. A total of 31 infants with dysontogenesis or chromosome abnormality were excluded. Those who could not been diagnosed whether they had HCA were not included (*n* = 21). Subjects who had twin and multiple premature infants were excluded (*n* = 156). Those with incomplete clinical data on maternal weight and height (*n* = 4), history of preterm birth (*n* = 3), gravidity (*n* = 1), maternal education level (*n* = 1), premature rupture time of membranes (*n* = 1), whether *in vitro* fertilization (IVF) was applied (*n* = 4), prenatal insulin use (*n* = 1), prenatal glucocorticoid use (*n* = 1), and prenatal maternal platelets (*n* = 1) were also excluded. Finally, 673 participants were included in this study. The detailed screen process was presented in Figure 1. All participants were divided into HCA group (*n* = 195) and non-HCA group (*n* = 478). The diagnosis of HCA was based on the results of pathology of placenta. The study was approved by the Ethics Committee of Northwest Women's and Children's Hospital (No. 2022-074), and all participants provided the informed consent form before enrollment.

### Data collection

The demographic and clinical data of the pregnant mothers were collected including age, body mass index (BMI, kg/cm^2^), history of preterm birth, gravidity, parity, frequency of abortion, education (below high school, senior high school, or university and above), premature rupture of membranes or not, IVF or not, hypertension or not, diabetes or not, prenatal insulin use or not, prenatal antibiotic use or not, prenatal glucocorticoid use (partial course, single course, multiple courses, or not used), prenatal use of magnesium sulfate or not, prenatal fever or not, duration of fever (h), increase of fetal heart rate or not, placental abruption or not, increase of maternal heart rate or not, placenta previa or not, white blood cells (WBC, 10^9^/L), neutrophil percentage (NEUT%), lymphocyte ratio (LYM%), neutrophil-to-lymphocyte ratio (NLR), hemoglobin (g/L), platelet (10^9^/L), CRP (<5, 5–10, 10–20, >20 mg/L), albumin (g/L), Group B streptococcus (GBS: yes, no, or not detected), vaginitis during pregnancy or not, oligohydramnios or not, complications of diabetes or hypertension or not, cholestasis or not, abnormal fetal heart rate or not, length of placenta, width of placenta, thickness of placenta, and fetal distress.

### Definition of the variables

Premature rupture of membranes was defined as the rupture of membranes before contractions. Premature rupture time of membranes indicated the time between the rupture of membranes and delivery. Oligohydramnios was identified based on prenatal B ultrasound with amniotic fluid index of ≤5 cm, total amniotic fluid volume of <300 ml during the third trimester, or maximum vertical depth of amniotic fluid in the third trimester as ≤2 cm. Placenta previa referred to the placenta attaching to the lower uterine segment and partially or completely covers the internal cervix based on ultrasound. The diagnosis of placental abruption was based on the clinical findings of abdominal pain, vaginal bleeding, uterine contractions, fetal distress, and vital sign abnormalities after 20 weeks of gestation. Intrahepatic cholestasis during pregnancy was defined as the fasting serum total bile acid of ≥10 μmol/L accompanied by skin itching and other symptoms during pregnancy.

### Outcome variables

The primary outcome in this study was the discrimination/AUC of the predictive model to identify HCA. HCA was mainly based on the results of placenta pathology calculating the number of Ne in the placenta and fetal membrane. The Ne count is classified as grade I when it reaches 5–10, grade II when it ranges from 11 to 30, and grade III for counts exceeding 30.

The secondary outcomes were BPD, necrotizing enterocolitis (NEC), sepsis, retinopathy of prematurity (ROP), brain damage, neonatal infection, or death in preterm newborns. Brain damage was diagnosed based on brain B ultrasound or brain magnetic resonance imaging after 72 h–96 h of birth. ROP was screened 4–6 weeks after birth. A diagnosis of BPD was made when the administration of oxygen at a fraction of inspired oxygen (FiO_2_) at >21 was required for a duration of 28 days or longer (16). NEC was defined as the inflammation of the small or large intestine with different degrees of severity according to Bell's criteria modified by Gutiérrez et al. (17). The diagnosis of sepsis was based on the abnormal clinical manifestations and in accordance with any of the following: (1) Pathogenic bacteria were found in blood culture or sterile cavity culture. (2) If the blood culture was a conditional pathogen, the same bacteria must be cultured with another blood sample or sterile cavity. The clinical diagnosis of sepsis was based on the abnormal clinical manifestations, with any of the following: (1) abnormal non-specific laboratory indicators of blood at ≥2. (2) Bacterial meningitis changes in cerebrospinal fluid examination. (3) DNA of pathogenic bacteria was detected in the blood.

### Binary logistic regression model

The predictors for the risk of HCA were screened via binary logistic regression model. All the variables were included in the model, and the stepwise method was used. Variables with statistical association with HCA were retained as the predictors. The binary logistic regression was used to construct the prediction model, and the associations between the predicted risk of HCA and the actual HCA patients and secondary outcomes were analyzed.

### Statistical analysis

Quantitative data with non-normal distribution were displayed as M [first quartile, third quartile (Q_1_, Q_3_)], and comparisons were conducted via Wilcoxon rank sum test. Qualitative data were shown as *n* (%), and the differences between groups were compared via chi-square test. Sensitivity analysis was performed to compare the data of the participants with and without placenta examination (Supplementary Table S1). The stepwise method was used to screen the predictors for HCA, the logistic regression was used to construct the prediction model, and the associations between the predicted risk of HCA and secondary outcomes were analyzed. In the adjusted model, gestational age and weight were adjusted. The best cut-off point was determined by the maximum Youden's index. The evaluation of the predictive value of the model was subjected to AUC, accuracy, sensitivity and specificity, negative predictive value (NPV), and positive predictive value (PPV). The odds ratio (OR) was applied to assess the association of predicted risk of HCA and secondary outcomes. The receiver operator characteristic curve (ROC) curve, Kolmogorov–Smirnov (KS), calibration curve, calibration curve, and nomogram of the prediction model were plotted. The confidence level was set as 0.05, and all analyses were performed using SAS9.4.

## Results

### Comparisons of the participants’ characteristics from HCA group and non-HCA group

In total, 673 participants were enrolled from the Northwest Women’s and Children's Hospital. Among them, 195 participants were in the HCA group, and 478 were in the non-HCA group. As displayed in Table 1, the proportions of participants with premature rupture of membranes (65.64% vs. 31.59%), prenatal antibiotic use (69.74% vs. 33.47%), and maternal CRP level of ≥20 mg/L (33.33% vs. 6.69%) in the HCA group were higher than in the non-HCA group. The mean NLR in the HCA group was higher than in the non-HCA group (6.72 vs. 4.46).

**Table 1:** Comparisons of the participants’ characteristics from HCA group and non-HCA group.

		Group			
Variables	Total (*n* = 673)	Non-HCA (*n* = 478)	HCA (*n* = 195)	Statistics	*P*-value
Mother age, years, mean ± SD	30.96 ± 4.35	30.83 ± 4.25	31.30 ± 4.60	*t* = −1.27	0.205
BMI, kg/m^2^, mean ± SD	25.62 ± 3.91	25.81 ± 4.01	25.16 ± 3.62	*t* = 1.96	0.051
History of preterm birth, M (Q_1_,Q_3_)	0.00 (0.00, 0.00)	0.00 (0.00, 0.00)	0.00 (0.00, 0.00)	*Z* = −1.03	0.305
Gravidity, M (Q_1_,Q_3_)	2.00 (1.00, 3.00)	2.00 (1.00, 3.00)	2.00 (1.00, 3.00)	*Z* = −0.24	0.814
Parity, M (Q_1_,Q_3_)	1.00 (1.00, 2.00)	1.00 (1.00, 2.00)	2.00 (1.00, 2.00)	*Z* = 1.34	0.180
Frequency of abortion, M (Q_1_,Q_3_)	1.00 (0.00, 2.00)	1.00 (0.00, 2.00)	1.00 (0.00, 1.00)	*Z* = −1.16	0.246
Education, *n* (%)				*χ*^2 ^= 0.024	0.877
Below senior high school	210 (31.20)	150 (31.38)	60 (30.77)		
University and above	463 (68.80)	328 (68.62)	135 (69.23)		
Premature rupture of membranes, *n* (%)				χ^2 ^= 66.17	<0.001
No	394 (58.54)	327 (68.41)	67 (34.36)		
Yes	279 (41.46)	151 (31.59)	128 (65.64)		
IVF, *n* (%)				χ^2 ^= 1.71	0.191
No	613 (91.08)	431 (90.17)	182 (93.33)		
Yes	60 (8.92)	47 (9.83)	13 (6.67)		
Hypertension, *n* (%)				χ^2 ^= 51.03	<0.001
No	511 (75.93)	327 (68.41)	184 (94.36)		
Yes	162 (24.07)	151 (31.59)	11 (5.64)		
Diabetes, *n* (%)				χ^2 ^= 0.01	0.913
No	523 (77.71)	372 (77.82)	151 (77.44)		
Yes	150 (22.29)	106 (22.18)	44 (22.56)		
Prenatal insulin use, *n* (%)				—	0.537
No	660 (98.07)	470 (98.33)	190 (97.44)		
Yes	13 (1.93)	8 (1.67)	5 (2.56)		
Prenatal antibiotic use, *n* (%)				χ^2 ^= 73.95	<0.001
No	377 (56.02)	318 (66.53)	59 (30.26)		
Yes	296 (43.98)	160 (33.47)	136 (69.74)		
Prenatal glucocorticoid use, *n* (%)				χ^2 ^= 5.39	0.145
Not used	86 (12.78)	56 (11.72)	30 (15.38)		
Partial course	206 (30.61)	150 (31.38)	56 (28.72)		
Single course	356 (52.90)	250 (52.30)	106 (54.36)		
Multiple courses	25 (3.71)	22 (4.60)	3 (1.54)		
Prenatal use of magnesium sulfate, *n* (%)				χ^2 ^= 0.25	0.619
No	220 (32.69)	159 (33.26)	61 (31.28)		
Yes	453 (67.31)	319 (66.74)	134 (68.72)		
Prenatal fever, *n* (%)				-	0.163
No	663 (98.51)	473 (98.95)	190 (97.44)		
Yes	10 (1.49)	5 (1.05)	5 (2.56)		
Duration of fever, hours, M (Q_1_,Q_3_)	0.00 (0.00, 0.00)	0.00 (0.00, 0.00)	0.00 (0.00, 0.00)	*Z* = 1.78	0.075
Increase of fetal heart rate, *n* (%)				—	1.000
No	668 (99.26)	474 (99.16)	194 (99.49)		
Yes	5 (0.74)	4 (0.84)	1 (0.51)		
Increase of maternal heart rate, *n* (%)				—	0.049
No	665 (98.81)	475 (99.37)	190 (97.44)		
Yes	8 (1.19)	3 (0.63)	5 (2.56)		
Placental abruption, *n* (%)				χ^2 ^= 6.40	0.011
No	617 (91.68)	430 (89.96)	187 (95.90)		
Yes	56 (8.32)	48 (10.04)	8 (4.10)		
Placenta previa, *n* (%)				χ^2 ^= 6.45	0.011
No	626 (93.02)	437 (91.42)	189 (96.92)		
Yes	47 (6.98)	41 (8.58)	6 (3.08)		
WBC, M (Q_1_,Q_3_)	10.12 (8.10, 12.59)	9.45 (7.66, 11.63)	11.88 (9.65, 14.43)	*Z* = 7.61	<0.001
NEUT%, mean ± SD	77.19 ± 9.24	75.87 ± 9.17	80.43 ± 8.63	*t* = −5.95	<0.001
LYM%, M (Q_1_,Q_3_)	15.70 (10.80, 20.90)	17.15 (12.20, 22.30)	12.10 (8.80, 16.50)	*Z* = −7.84	<0.001
NLR, M (Q_1_,Q_3_)	4.97 (3.40, 7.61)	4.46 (3.15, 6.74)	6.72 (4.60, 9.82)	*Z* = 7.63	<0.001
Hemoglobin, mean ± SD	115.51 ± 26.09	117.45 ± 27.22	110.74 ± 22.45	*t* = 3.30	0.001
Platelet, mean ± SD	196.28 ± 60.28	192.58 ± 62.30	205.36 ± 54.09	*t* = −2.66	0.008
CRP, *n* (%)				χ^2 ^= 139.56	<0.001
<5	375 (55.72)	321 (67.15)	54 (27.69)		
5–10	121 (17.98)	91 (19.04)	30 (15.38)		
10–20	80 (11.89)	34 (7.11)	46 (23.59)		
>20	97 (14.41)	32 (6.69)	65 (33.33)		
Albumin, mean ± SD	36.41 ± 6.09	36.58 ± 6.31	35.96 ± 5.49	*t* = 1.24	0.215
GBS, *n* (%)				χ^2 ^= 39.97	<0.001
No	388 (57.65)	240 (50.21)	148 (75.90)		
Yes	11 (1.63)	8 (1.67)	3 (1.54)		
No	274 (40.71)	230 (48.12)	44 (22.56)		
Vaginitis during pregnancy, *n* (%)				χ^2 ^= 0.00	0.953
No	631 (93.76)	448 (93.72)	183 (93.85)		
Yes	42 (6.24)	30 (6.28)	12 (6.15)		
Oligohydramnios, *n* (%)				χ^2 ^= 2.15	0.142
No	551 (81.87)	398 (83.26)	153 (78.46)		
Yes	122 (18.13)	80 (16.74)	42 (21.54)		
Cholestasis, *n* (%)				χ^2 ^= 8.85	0.003
No	640 (95.10)	447 (93.51)	193 (98.97)		
Yes	33 (4.90)	31 (6.49)	2 (1.03)		
Abnormal fetal heart rate, *n* (%)				χ^2 ^= 5.88	0.015
No	642 (95.39)	450 (94.14)	192 (98.46)		
Yes	31 (4.61)	28 (5.86)	3 (1.54)		
Length of placenta, mean ± SD	15.64 ± 2.62	15.60 ± 2.70	15.74 ± 2.39	*t* = −0.68	0.498
Width of placenta, mean ± SD	14.65 ± 3.60	14.57 ± 3.65	14.85 ± 3.50	*t* = −0.90	0.366
Thickness of placenta, M (Q_1_,Q_3_)	3.00 (2.50, 5.00)	3.00 (2.50, 5.00)	3.00 (2.50, 5.00)	*Z* = 0.491	0.623
Fetal distress, *n* (%)				χ^2 ^= 15.82	<0.001
No	598 (88.86)	410 (85.77)	188 (96.41)		
Yes	75 (11.14)	68 (14.23)	7 (3.59)		
Gestational age, weeks, mean ± SD	31.70 ± 2.07	32.00 ± 1.58	30.97 ± 2.82	*t* = 4.81	<0.001
Birth weight, g, mean ± SD	1,691.37 ± 424.09	1,708.39 ± 413.71	1,649.74 ± 446.83	*t* = 1.63	0.104

### Construction of the logistic prediction model for HCA

**Figure 2:**
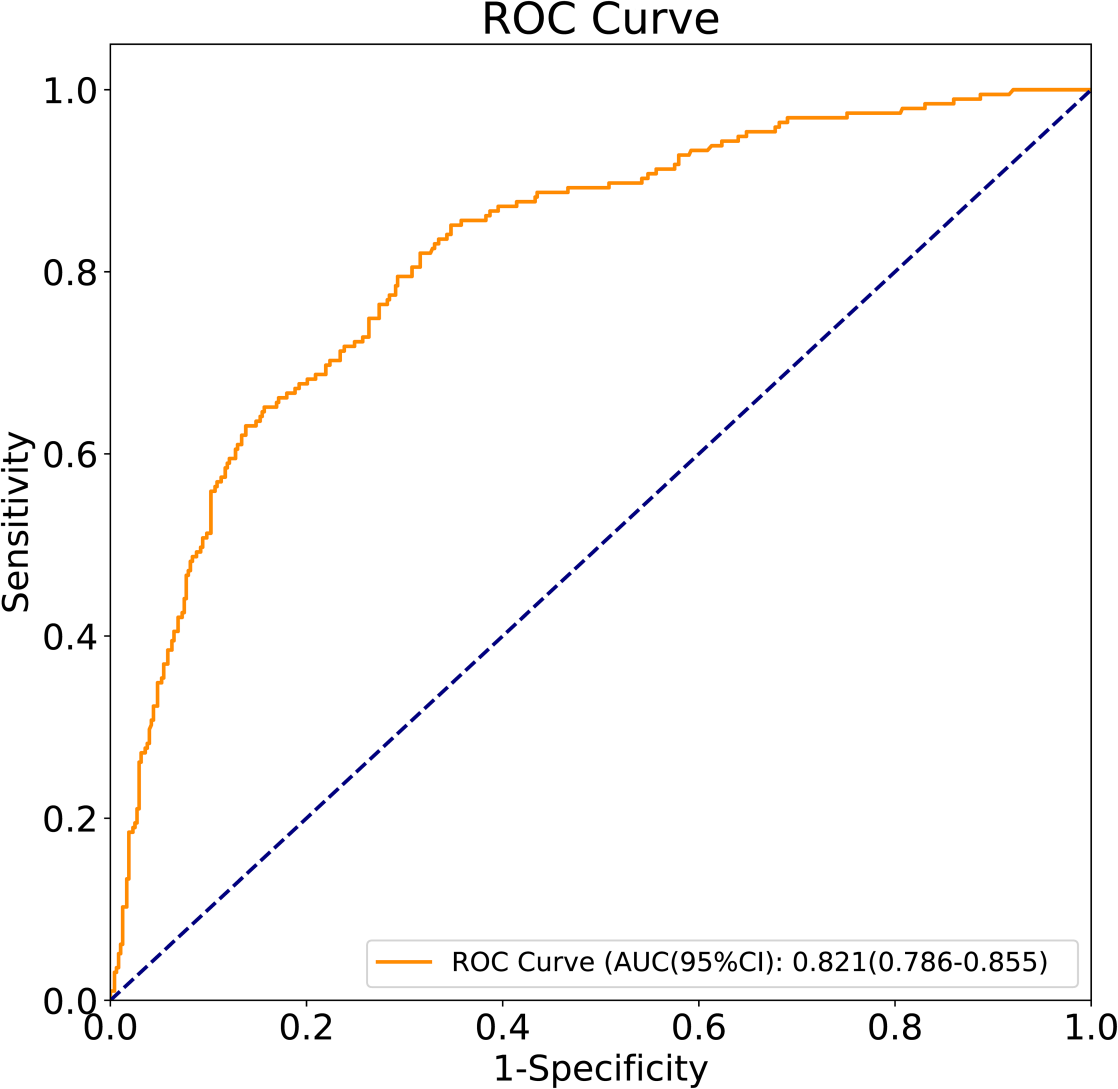
ROC curve of the prediction model.

**Figure 3:**
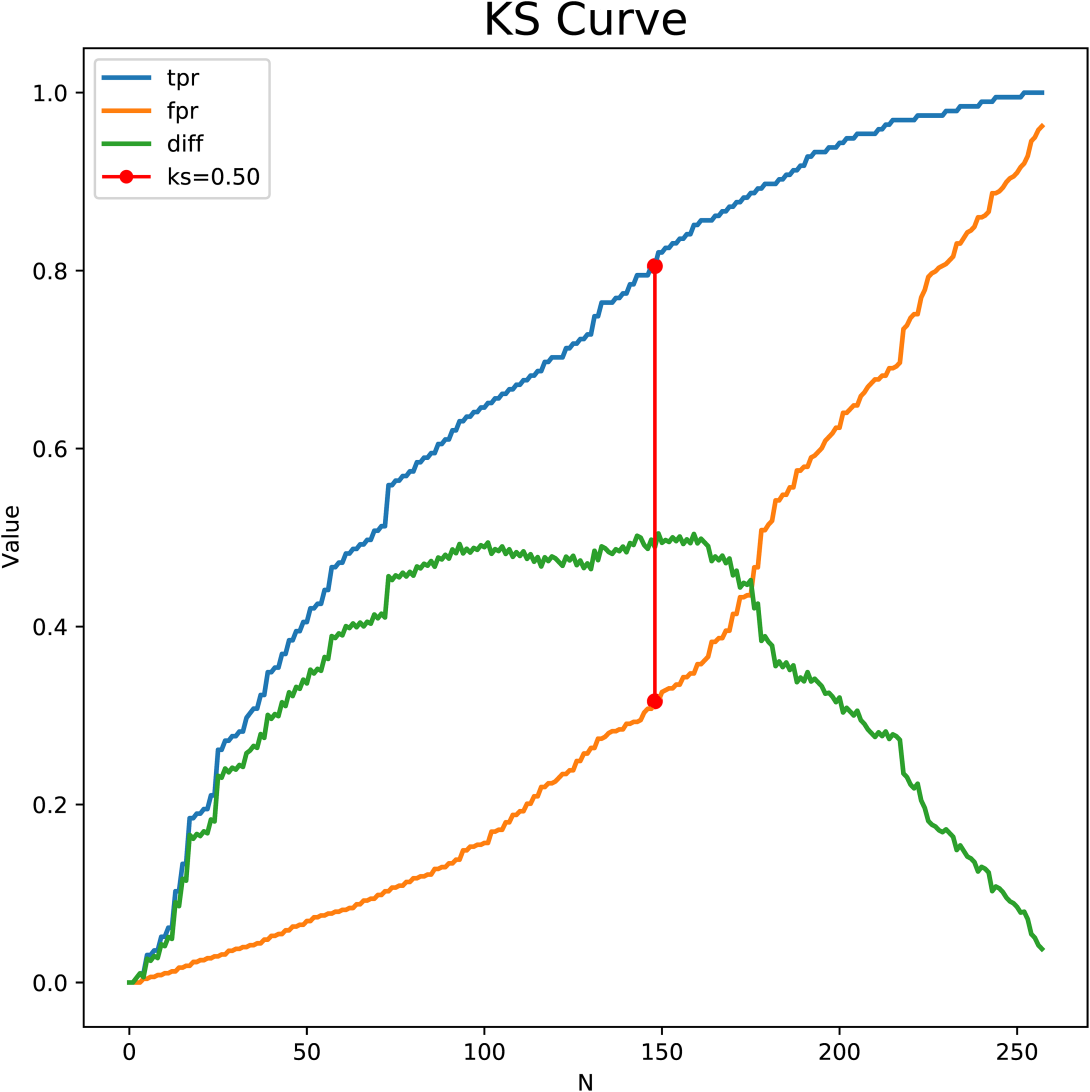
KS curve of the prediction model.

**Figure 4:**
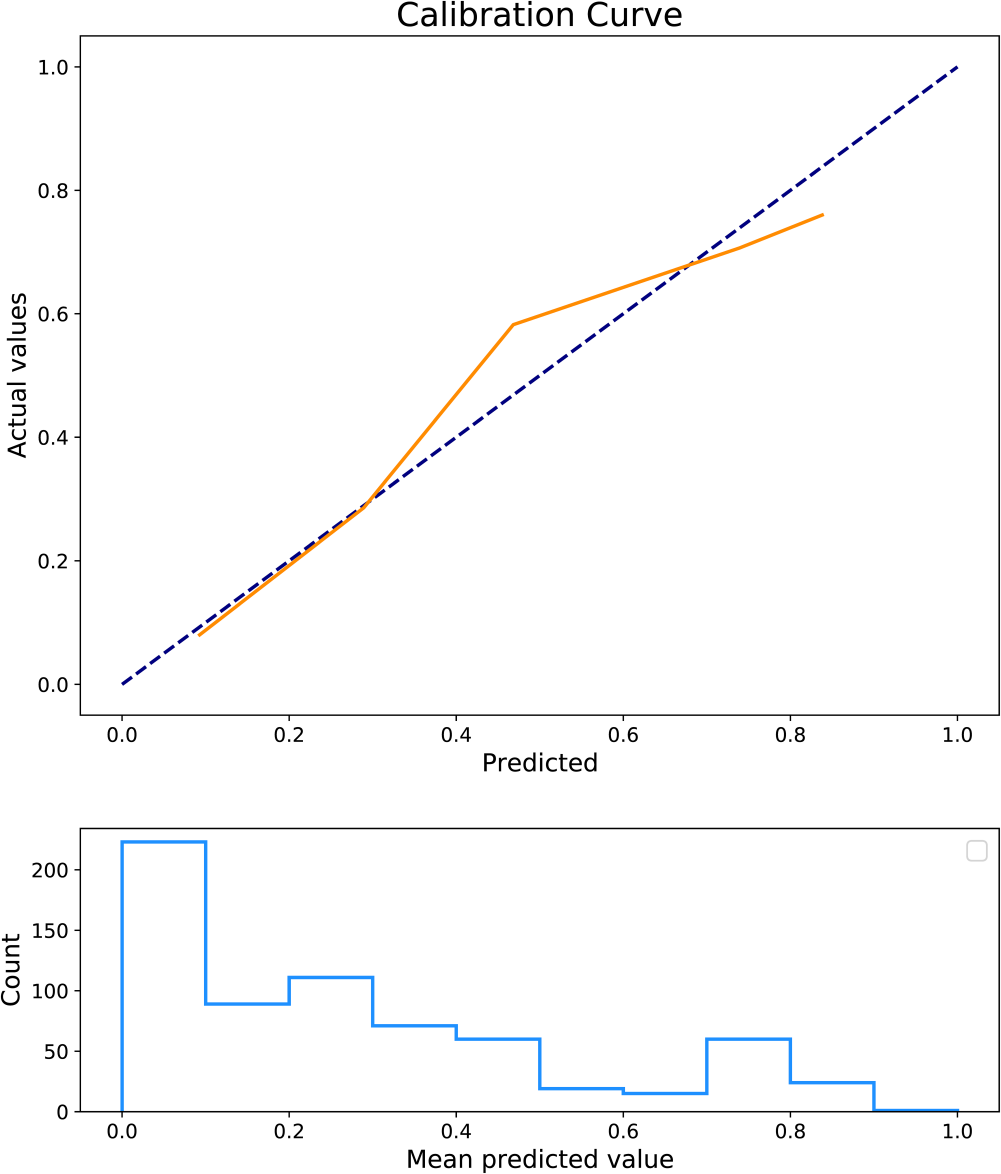
Calibration curve of the prediction model.

**Figure 5:**
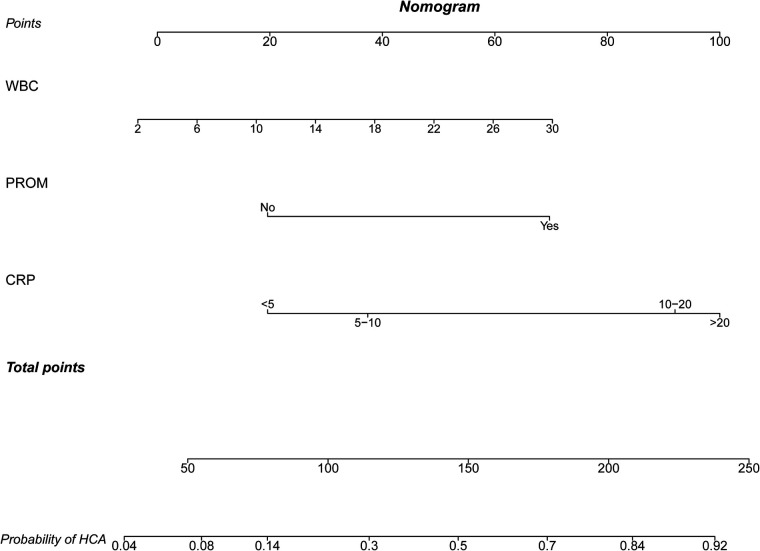
Nomogram for the prediction model.

The predictors for HCA were screened via the binary logistic stepwise regression. From Table 2, we observed that premature rupture of membranes (OR = 3.81, 95% CI: 2.56–5.67), WBC (OR = 1.07, 95% CI: 1.02–1.13), CRP of 10–20 (OR = 6.92, 95% CI: 3.92–12.23), and CRP of >20 (OR = 8.56, 95% CI: 4.87–15.04) were associated with the risk of HCA in mothers. The final prediction model was Logit (P) = −3.096 + 1.339 × premature rupture of membranes (yes) + 0.070 × WBC + 0.475 × CRP (5–10) + 1.935 × CRP (10–20) + 2.147 × CRP (>20). The sensitivity of the prediction model was 0.821 (95% CI: 0.767–0.874), the specificity was 0.684 (95% CI: 0.642–0.726), the PPV was 0.514 (0.459–0.570), the NPV was 0.903 (95% CI: 0.873–0.934), the AUC was 0.821 (95% CI: 0.786–0.855), and the accuracy was 0.724 (95% CI: 0.690–0.757). The ROC curve of the model was exhibited in Figure 2. The KS curve (Figure 3) and calibration curve (Figure 4) indicated that the model had good predictive performance. In addition, the nomogram of the prediction model was presented in Figure 5.

**Table 2:** Construction of the logistic prediction model for HCA.

Variable	*β*	Standard error	OR (95% CI)	*P-*value
Constant	−3.096	0.337		<0.001
Premature rupture of membranes				
No			Ref	
Yes	1.339	0.203	3.81 (2.56–5.67)	<0.001
WBC	0.070	0.027	1.07 (1.02–1.13)	0.010
CRP				
<5			Ref	
5–10	0.475	0.271	1.61 (0.95–2.73)	0.079
10–20	1.935	0.291	6.92 (3.92–12.23)	<0.001
>20	2.147	0.288	8.56 (4.87–15.04)	<0.001

### Associations between the predicted risk of HCA and adverse outcomes in preterm infants

According to the data in Table 3, the risk of BPD in preterm infants was increased as the increase of the predicted risk of HCA (OR = 3.48, 95% CI: 1.10–10.95). The increase of the predicted risk of HCA was associated with an elevated risk of sepsis (OR = 6.66, 95% CI: 2.17–20.43). A higher risk of ROP was observed in infants with a higher predicted risk of HCA (OR = 1.59, 95% CI: 0.37–6.85). The predicted risk of HCA was correlated with an increased risk of neonatal infections (OR = 9.85, 95% CI: 3.59–26.98).

**Table 3:** Associations between the predicted risk of HCA and adverse outcomes in preterm infants.

Dependent variable	*N*	*Β*	Standard error	OR (95% CI)	*P-*value
BPD					
No	628			Ref	
Yes	45	1.247	0.585	3.48 (1.10–10.95)	0.033
NEC					
No	595			Ref	
Yes	78	−0.393	0.524	0.67 (0.24–1.88)	0.452
Sepsis					
No	628			Ref	
Yes	45	1.897	0.571	6.66 (2.17–20.43)	<0.001
ROP					
<2	643			Ref	
≥2	30	0.463	0.746	1.59 (0.37–6.85)	0.534
Brain damage					
No	541			Ref	
Yes	131	0.571	0.394	1.77 (0.82–3.83)	0.147
Neonatal infection					
No	613			Ref	
Yes	57	2.287	0.514	9.85 (3.59–26.98)	<0.001
Neonatal death					
No	648			Ref	
Yes	25	0.407	0.817	1.50 (0.30–7.45)	0.619

### Associations between HCA and adverse outcomes in preterm infants

The associations between HCA and adverse outcomes in preterm infants were further analyzed. In the unadjusted model, the results demonstrated that patients with HCA were associated with a higher risk of BPD in preterm infants (OR = 2.77, 95% CI: 1.51–5.10). An elevated risk of sepsis in preterm infants was also identified in those diagnosed with HCA (OR = 2.07, 95% CI: 1.12–3.82). The risk of neonatal infections in preterm infants was increased in patients with HCA (OR = 6.40, 95% CI: 3.56–11.52). After adjusting for confounders including gestational week at birth and birth weight, the risk of neonatal infections (OR = 5.03, 95% CI: 2.69–9.41) was also increased in preterm infants’ exposure to HCA (Table 4).

**Table 4:** Associations between HCA and adverse outcomes in preterm infants.

Dependent variable	*N*	Unadjusted model		Adjusted model^a^	
		OR (95% CI)	*P*-value	OR (95% CI)	*P*-value
BPD					
No	628	Ref		Ref	
Yes	45	2.77 (1.51–5.10)	0.001	1.24 (0.59–2.62)	0.573
NEC					
No	595	Ref		Ref	
Yes	78	0.20 (0.10–0.41)	<0.001	0.57 (0.31–1.04)	0.066
Sepsis					
No	628	Ref		Ref	
Yes	45	2.07 (1.12–3.82)	0.020	1.54 (0.75–3.15)	0.240
ROP					
<2	643	Ref		Ref	
≥2	30	1.94 (0.92–4.07)	0.081	0.81 (0.33–2.01)	0.649
Brain damage					
No	541	Ref		Ref	
Yes	131	1.10 (0.73–1.67)	0.639	1.00 (0.64–1.56)	0.996
Neonatal infection					
No	613	Ref		Ref	
Yes	57	6.40 (3.56–11.52)	<0.001	5.03 (2.69–9.41)	<0.001
Neonatal death					
No	648	Ref		Ref	
Yes	25	1.98 (0.88–4.44)	0.097	1.21 (0.47–3.15)	0.693

^a^ Adjusting gestational week at birth and birth weight.

## Discussion

This study collected the data of 673 subjects who delivered in Northwest Women’s and Children's Hospital for constructing a prediction model for HCA and evaluating the association between the predicted risk of HCA and the adverse outcomes in preterm infants as well as the risk of adverse outcomes in preterm infants in women with HCA. The prediction model showed good predictive performance for HCA. The predicted risk of HCA was associated with the risk of BPD, sepsis, and neonatal infections. HCA was associated with a higher risk of infections in infants. The findings of our study might quickly help identify those with a high risk of HCA, provide effective interventions on preventing the occurrence of HCA, and improve the outcomes of these patients.

Previously, the premature rupture of membranes was identified to be a risk factor for HCA (18). HCA patients with premature rupture of membranes were associated with an increased risk of poor neonatal outcomes (19). This gave support to the results of our study, which revealed that premature rupture of membranes was an important predictor for HCA in pregnant women. In addition, the maternal CRP was another vital predictor for HCA in pregnant women. Serum CRP concentration ranges from 5 to 10 mg/L in healthy individuals but increases to 10–40 mg/L among pregnant women with mild inflammation and >40 mg/L in conditions such as moderate to severe inflammation (20). Maternal CRP was considered to be an accurate predictor of CA (21). This was allied with the results of our study. This may be because cytokines including interleukin 1, interleukin 6, and tumor necrosis factor alpha can induce acute-phase proteins in the liver such as CRP in infections and other inflammatory states (22). WBC was higher in pregnant women with HCA (23), and we also identified that WBC might be a reliable predictor for HCA.

The logistic prediction model for HCA was constructed based on the data of 684 participants using the predictors including premature rupture of membranes, WBC, and maternal CRP levels. Previous prediction models were applied for identifying patients with a high risk of HCA in those with premature rupture of membranes (12, 13, 24–26). Premature rupture of membranes in pregnant women is not usual, which might increase the attention of obstetricians on those patients (27). It is itself also a predictor for HCA (18). However, many HCA patients were not complicated with premature rupture of membranes, and HCA in those patients was more difficult to identify. A prediction model to quickly identify HCA in those patients is essential, and the prediction model in our study had wider applicability. In addition, compared with previous prediction models, the predictive performance in the current study including the AUC, sensitivity, specificity, NPV, and accuracy was very good, which indicated that our model had good predictive ability. In addition, the nomogram of the prediction model was plotted, which could easily calculate the score directly from the graph, and obtained the probability of HCA in patients. The prediction model might offer an easy and useful clinical tool for identifying those with a high risk of HCA. For patients who were predicted to have a high risk of HCA, further examination should be performed to timely diagnose HCA and provide appropriate treatments and special care. Early treatment of reproductive tract infections such as trichomoniasis vaginitis, Neisseria gonorrhea infection, cervical chlamydia trachomatis infection, bacterial vaginosis, and other diseases, including urinary tract infections such as asymptomatic bacteriuria, should be treated as soon as possible and actively treated according to medical advice. More trace elements such as copper and zinc and vitamin C should be supplemented to decrease tension and rupture of the fetal membrane. Appropriate antibiotics should be applied in those who had premature rupture of membranes, as antibiotic use was reported to potentially prevent a possible ascending infection, reduce the incidence of HCA, and improve neonatal outcomes (28). A meta-analysis by Alrowaily et al. recommended that for patients who were diagnosed with CA, well-designed trials using standard definitions of CA, outcome measures, and newer antibiotics were required to inform a clinical practice for choosing the preferred antibiotic regimen, dose, and duration to optimize maternal and neonatal outcomes (29). Herein, HCA was found to be associated with higher risks of infections in infants, which was similar to the results of the predicted risk of HCA and infections, indicating the necessity and essential of early prediction of HCA.

In China, some women would like to bring their placenta home and deal with it by themselves according to their custom. They did not want to exam their placenta. Some of the HCA cases might be missed. Moreover, the results of placenta pathology were available approximately 3 days in some hospital, and this might cause a delay of the treatment of infants. For infants with predicted high risk of HCA exposure, the evaluation should be done in a timely manner, and timely treatments should be provided. Our prediction model using non-invasive biological markers, which are to use, and their results are readily available.

In the current study, a higher risk of HCA was associated with an elevated risk of BPD. This was allied by a meta-analysis by Villamor-Martinez, indicating that exposure to CA was associated with a higher risk of BPD in preterm infants (30). CA-induced increased concentrations of inflammatory factors such as IL-1, IL-6, and IL-8 may also aggravate the pulmonary inflammation via increasing pulmonary vascular resistance, hindering alveolar development, accelerating pulmonary fibrosis, and ultimately leading to BPD (31). In the present study, after adjusting for gestational week at birth and birth weight, HCA was not associated with the risk of BPD in preterm infants; this may be because the incidence of BPD was linked to the gestational week at birth and birth weight. Infants with a smaller gestational age and lower birth weight were correlated with a higher risk of BPD (32). The association of NRDS with HCA has been identified in previous studies. Ecevit et al. found that 76.1% of infants had NRDS in positive HCA group and the incidence of NRDS was correlated with HCA (33). This was allied with the results in the current study, showing that a higher risk of NRDS was observed in both mothers with a higher predicted risk of HCA and those who had HCA. Recently, Beck et al. conducted a meta-analysis and found that there was an association between HCA and the risk of infections or even early- and late-onset sepsis in neonates (4). This supported the finding in this study, which found that higher predicted risk of HCA was linked to a higher risk of infections or sepsis in neonates. In addition, in those who were diagnosed with HCA, the risk of infections was also increased. This may be because exposure to HCA might affect the monocyte transcriptional response to the neonatal pathogen *Staphylococcus epidermidis* and increase the risk of sepsis in neonates (34).

Currently, for HCA-exposed infants, empirical antibiotic therapy was recommended as a guideline for the treatment of these patients, but some clinicians question these recommendations due to the potential antibiotic risks (35). Another recent study also demonstrated that asymptomatic full-term infants born to women with CA may not need routine antibiotics (36). In the present study, in infants with a high risk of HCA, we found that the risks of BPD, NRDS, sepsis, and infections were still high although antibiotics were applied, which suggested that there might be misuse of antibiotics in these participants. For pediatricians, for mothers predicted with a low risk of HCA and asymptomatic infants, the use of antibiotics should be carefully applied.

Several limitations in our study were reported. First, this was a retrospective cohort study, and the information on the participants might have bias. Second, no external validation was performed to verify the prediction model in this study. Third, there might be missing data bias, as not every placenta was examined in our participants. In the future, well-designed prospective studies with a large scale of sample size are required to validate the result in the current study.

## Conclusion

The data of 673 subjects who delivered in Northwest Women’s and Children's Hospital were used to establish a prediction model for HCA in this study. The model showed good predictive performance for identifying pregnant women with a higher risk of HCA. In addition, the predicted risk of HCA was associated with the risk of BPD, sepsis, and infections in neonates. The findings of our study might help early identify those who have a high risk of HCA and provide timely interventions to improve the outcomes of infants.

## Data Availability

The raw data supporting the conclusions of this article will be made available by the authors, without undue reservation.
