# Supplementary material for: Establishment of a prediction model for histological chorioamnionitis and its association with outcomes of premature infants
Source: Front Pediatr. 2023 Aug 16;11:1194563. doi: 10.3389/fped.2023.1194563 (PMC10466889; doi:10.3389/fped.2023.1194563)
Supplement: Supplementary file 1 [file Table1.docx]

Supplementary Table 1 Sensitivity analysis of the baseline data of women with and without placenta examination

| Variables | Total (n=1101) | Women with placenta examination (n=673) | Women without placenta examination (n=428) | Statistics | *P* |
| --- | --- | --- | --- | --- | --- |
| Mother age, years, Mean ± SD | 30.86 ± 4.27 | 30.96 ± 4.35 | 30.70 ± 4.14 | t=1.00 | 0.319 |
| BMI, kg/m^2^, M(Q_1_,Q_3_) | 25.39 (22.96,28.07) | 25.10 (22.86,28.23) | 25.59 (23.24,28.04) | Z=1.597 | 0.110 |
| History of preterm birth, M(Q_1_,Q_3_) | 0.00 (0.00,0.00) | 0.00 (0.00,0.00) | 0.00 (0.00,0.00) | Z=0.293 | 0.769 |
| Gravidity, M(Q_1_,Q_3_) | 2.00 (1.00,3.00) | 2.00 (1.00,3.00) | 2.00 (1.00,3.00) | Z=0.023 | 0.982 |
| Parity, M(Q_1_,Q_3_) | 1.00 (1.00,2.00) | 1.00 (1.00,2.00) | 1.00 (1.00,2.00) | Z=-0.303 | 0.762 |
| Frequency of abortion, M(Q_1_,Q_3_) | 1.00 (0.00,2.00) | 1.00 (0.00,2.00) | 1.00 (0.00,2.00) | Z=0.242 | 0.808 |
| Education, n (%) |  |  |  | χ^2^=1.443 | 0.230 |
| Below senior high school | 329 (29.88) | 210 (31.20) | 119 (27.80) |  |  |
| University and above | 772 (70.12) | 463 (68.80) | 309 (72.20) |  |  |
| Premature rupture of membranes, n (%) |  |  |  | χ^2^=0.421 | 0.517 |
| No | 653 (59.31) | 394 (58.54) | 259 (60.51) |  |  |
| Yes | 448 (40.69) | 279 (41.46) | 169 (39.49) |  |  |
| IVF, n (%) |  |  |  | χ^2^=10.225 | 0.001 |
| No | 976 (88.65) | 613 (91.08) | 363 (84.81) |  |  |
| Yes | 125 (11.35) | 60 (8.92) | 65 (15.19) |  |  |
| Hypertension, n (%) |  |  |  | χ^2^=0.758 | 0.384 |
| No | 826 (75.02) | 511 (75.93) | 315 (73.60) |  |  |
| Yes | 275 (24.98) | 162 (24.07) | 113 (26.40) |  |  |
| Diabetes, n (%) |  |  |  | χ^2^=0.106 | 0.745 |
| No | 852 (77.38) | 523 (77.71) | 329 (76.87) |  |  |
| Yes | 249 (22.62) | 150 (22.29) | 99 (23.13) |  |  |
| Prenatal insulin use, n (%) |  |  |  | χ^2^=158.354 | <0.001 |
| No | 973 (88.37) | 660 (98.07) | 313 (73.13) |  |  |
| Yes | 128 (11.63) | 13 (1.93) | 115 (26.87) |  |  |
| Prenatal antibiotic use, n (%) |  |  |  | χ^2^=28.602 | <0.001 |
| No | 546 (49.59) | 377 (56.02) | 169 (39.49) |  |  |
| Yes | 555 (50.41) | 296 (43.98) | 259 (60.51) |  |  |
| Prenatal glucocorticoid use, n (%) |  |  |  | χ^2^=110.856 | <0.001 |
| Not use | 237 (21.53) | 86 (12.78) | 151 (35.28) |  |  |
| Partial course | 347 (31.52) | 206 (30.61) | 141 (32.94) |  |  |
| Single course | 466 (42.33) | 356 (52.90) | 110 (25.70) |  |  |
| Multiple courses | 51 (4.63) | 25 (3.71) | 26 (6.07) |  |  |
| Prenatal use of magnesium sulfate, n (%) |  |  |  | χ^2^=0.167 | 0.683 |
| No | 365 (33.15) | 220 (32.69) | 145 (33.88) |  |  |
| Yes | 736 (66.85) | 453 (67.31) | 283 (66.12) |  |  |
| Prenatal fever, n (%) |  |  |  | χ^2^=21.426 | <0.001 |
| No | 1062 (96.46) | 663 (98.51) | 399 (93.22) |  |  |
| Yes | 39 (3.54) | 10 (1.49) | 29 (6.78) |  |  |
| Duration of fever, hours, M (Q_1_,Q_3_) | 0.00 (0.00,0.00) | 0.00 (0.00,0.00) | 0.00 (0.00,0.00) | Z=4.247 | <0.001 |
| Increase of fetal heart rate, n (%) |  |  |  | χ^2^=15.336 | <0.001 |
| No | 1078 (97.91) | 668 (99.26) | 410 (95.79) |  |  |
| Yes | 23 (2.09) | 5 (0.74) | 18 (4.21) |  |  |
| Increase of maternal heart rate, n (%) |  |  |  | χ^2^=11.556 | <0.001 |
| No | 1074 (97.55) | 665 (98.81) | 409 (95.56) |  |  |
| Yes | 27 (2.45) | 8 (1.19) | 19 (4.44) |  |  |
| Placental abruption, n (%) |  |  |  | χ^2^=0.007 | 0.933 |
| No | 1010 (91.73) | 617 (91.68) | 393 (91.82) |  |  |
| Yes | 91 (8.27) | 56 (8.32) | 35 (8.18) |  |  |
| Placenta previa, n (%) |  |  |  | χ^2^=2.397 | 0.122 |
| No | 1013 (92.01) | 626 (93.02) | 387 (90.42) |  |  |
| Yes | 88 (7.99) | 47 (6.98) | 41 (9.58) |  |  |
| WBC, M(Q_1_,Q_3_) | 11.03 (8.75,13.73) | 10.12 (8.10,12.59) | 12.55 (9.90,14.67) | Z=9.618 | <0.001 |
| NEUT%, Mean ± SD | 77.89 ± 9.19 | 77.19 ± 9.24 | 79.01 ± 9.00 | t=-3.19 | 0.001 |
| LYM%, M(Q_1_,Q_3_) | 14.80 (10.20,19.30) | 15.70 (10.80,20.90) | 13.70 (9.60,16.90) | Z=-5.415 | <0.001 |
| NLR, M(Q_1_,Q_3_) | 5.37 (3.81,8.21) | 4.97 (3.40,7.61) | 5.91 (4.47,8.89) | Z=5.303 | <0.001 |
| Hemoglobin, Mean ± SD | 113.12 ± 22.60 | 115.51 ± 26.09 | 109.27 ± 14.61 | t=5.06 | <0.001 |
| Platelet, Mean ± SD | 197.00 (158.00,237.00) | 194.00 (157.00,228.00) | 202.00 (162.00,259.00) | Z=2.560 | 0.010 |
| CRP, n (%) |  |  |  | χ^2^=168.755 | <0.001 |
| <5 | 485 (44.05) | 375 (55.72) | 110 (25.70) |  |  |
| 5-10 | 171 (15.53) | 121 (17.98) | 50 (11.68) |  |  |
| 10-20 | 143 (12.99) | 80 (11.89) | 63 (14.72) |  |  |
| >20 | 302 (27.43) | 97 (14.41) | 205 (47.90) |  |  |
| Albumin, Mean±SD | 34.33 ± 6.00 | 36.41 ± 6.09 | 30.50 ± 3.37 | t=19.62 | <0.001 |
| GBS, n(%) |  |  |  | χ^2^=7.500 | 0.024 |
| No | 603 (54.77) | 388 (57.65) | 215 (50.23) |  |  |
| Yes | 15 (1.36) | 11 (1.63) | 4 (0.93) |  |  |
| No | 483 (43.87) | 274 (40.71) | 209 (48.83) |  |  |
| Vaginitis during pregnancy, n (%) |  |  |  | χ^2^=7.693 | 0.006 |
| No | 1048 (95.19) | 631 (93.76) | 417 (97.43) |  |  |
| Yes | 53 (4.81) | 42 (6.24) | 11 (2.57) |  |  |
| Oligohydramnios, n (%) |  |  |  | χ^2^=0.276 | 0.599 |
| No | 896 (81.38) | 551 (81.87) | 345 (80.61) |  |  |
| Yes | 205 (18.62) | 122 (18.13) | 83 (19.39) |  |  |
| Cholestasis, n (%) |  |  |  | χ^2^=0.835 | 0.361 |
| No | 1052 (95.55) | 640 (95.10) | 412 (96.26) |  |  |
| Yes | 49 (4.45) | 33 (4.90) | 16 (3.74) |  |  |
| Abnormal fetal heart rate, n (%) |  |  |  | χ^2^=25.838 | <0.001 |
| No | 1014 (92.10) | 642 (95.39) | 372 (86.92) |  |  |
| Yes | 87 (7.90) | 31 (4.61) | 56 (13.08) |  |  |
| Length of placenta, Mean±SD | 15.77 ± 2.79 | 15.64 ± 2.62 | 15.98 ± 3.04 | t=-1.89 | 0.059 |
| Width of placenta, Mean±SD | 14.73 ± 3.38 | 14.65 ± 3.60 | 14.85 ± 2.97 | t=-1.00 | 0.318 |
| Thickness of placenta, M(Q_1_,Q_3_) | 2.50 (2.00,5.00) | 3.00 (2.50,5.00) | 2.00 (2.00,2.00) | Z=-23.804 | <0.001 |
| Fetal distress, n (%) |  |  |  | χ^2^=0.905 | 0.342 |
| No | 986 (89.55) | 598 (88.86) | 388 (90.65) |  |  |
| Yes | 115 (10.45) | 75 (11.14) | 40 (9.35) |  |  |
| Gestational age, weeks, Mean ± SD | 31.63 ± 2.00 | 31.70 ± 2.07 | 31.52 ± 1.89 | t=1.52 | 0.128 |
| Birth weight, g, Mean ± SD | 1672.77 ± 432.59 | 1691.37 ± 424.09 | 1643.57 ± 444.53 | t=1.79 | 0.074 |

BMI: body mass index, IVF: in vitro fertilization, WBC: white blood cells, NEUT%: neutrophil percentage, LYM%: lymphocyte ratio, NLR: neutrophil to lymphocyte ratio CRP: C-reactive protein, GBS: Group B streptococcus
